# Screening Method and Antibacterial Activity of 1,3,4-Oxadiazole Sulfone Compounds against Citrus Huanglongbing

**DOI:** 10.3390/ijms241310515

**Published:** 2023-06-22

**Authors:** Xin Luo, Yong Zhang, Xing Liu, Yue Zou, Hongyi Song, Sheng Wang, Jixiang Chen

**Affiliations:** National Key Laboratory of Green Pesticide, Key Laboratory of Green Pesticide and Agricultural Bioengineering, Ministry of Education, Center for R&D of Fine Chemicals of Guizhou University, Guiyang 550025, China; 17601460021@163.com (X.L.); zhangyong001103@163.com (Y.Z.); 15721762850@163.com (X.L.); zy15186251853@163.com (Y.Z.); shy2637649950@163.com (H.S.); swang3004@163.com (S.W.)

**Keywords:** Citrus Huanglongbing, rapid screening method, antibacterial activity, antibacterial agent

## Abstract

Citrus Huanglongbing (HLB) is one of the most destructive diseases in the citrus industry. At present, *Candidatus* Liberibacter asiaticus (*C*Las) cannot be cultured in vitro, and there is a lack of rapid methods to test antibacterial activity, which greatly hinders the discovery of new antibacterial agents against HLB. To establish a rapid screening method for antibacterial agents against HLB with simple operation, a short cycle, and a large number of tests, the *C*Las contents in leaves from different citrus branches, different leaves from the same citrus branch, and two halves of the same citrus leaf were detected. Compared with the leaves on different branches and different leaves on the same branch, the difference in *C*Las content of the left and right halves of the same leaf was small; the difference was basically between 0.7 and 1.3. A rapid and efficient method for primary screening agents against HLB termed the “half-leaf method” was established through our long-term optimization and improvement. To verify the stability and reliability of the activity data measured using this method, 6-chloropurine riboside, which is highly soluble in water, was used as the test agent, and its antibacterial activity against HLB was tested 45 times. The results of the antibacterial activity test showed little difference in the mean values of each data group, indicating that this method could be used as a rapid method for screening agents against HLB. We used this method to test the antibacterial activity of compounds synthesized by our research group against HLB and found that some of the compounds showed good activity.

## 1. Introduction

Citrus Huanglongbing (HLB), also known as yellow shoot disease, yellow blight, or green fruit disease, is a systemic disease caused by infection with *Candidatus* Liberibacter [1]. *Candidatus* Liberibacter can be divided into three species according to geographical location, which are *Candidatus* Liberibacter asiaticus (*C*Las), *Candidatus* Liberibacter africanus (*C*Laf), and *Candidatus* Liberibacter americanus (*C*Lam) [2,3,4]. *C*Las is the most widely distributed and the cause of the greatest damage because it is heat-resistant [5]. HLB was first reported in southern China in 1919 [3]. According to statistics, HLB has been diagnosed in 51 of the 140 citrus-producing countries [6]. The large-scale spread of HLB cannot be achieved without the help of *Diphorina citri* Kuwayama (ACP); the ACP is a major medium that acquires *C*Las by eating infected citrus and transmits it to healthy trees [7,8]. HLB poses a serious threat to citrus growth and causes huge economic losses. For example, in the Jiangxi Province of China, more than 50 million diseased trees have been cut down since 2013, with a direct economic loss of more than CNY 9 billion [9]. In Florida, USA, HLB caused losses of USD 3.63 billion between 2006 and 2012 and resulted in the loss of 6611 jobs [10].

Citrus plants infected with *C*Las usually show symptoms such as poor trunk development, root rot, thin canopy, yellow stems, spots on leaves, and overall plant decay [11]. The mottled yellow mosaic appearance can be used as an important basis for identifying HLB [12]. However, due to the long incubation period of *C*Las, the appearance of symptoms means that it has reached the point of serious illness. Therefore, PCR-based detection of HLB is the most important detection method [13]. On the other hand, *C*Las cannot be cultured in vitro, and there is a problem of uneven distribution of infection, which seriously hinders the biological research of *C*Las, and there is no effective method for screening anti-HLB agents [14,15].

The current measures to control and manage HLB are limited to control from the source and the spread, including planting disease-free seedlings, digging up symptomatic trees, and reducing the spread of *C*Las by controlling the number of ACPs [16]. Planting disease-free seedlings and then controlling the ACP media can effectively control the spread of HLB, but it cannot fundamentally prevent the disease. The cost of citrus production is high, and the period is prolonged by removing diseased plants and replanting disease-free seedlings after infection. It has been reported that 40–42 °C exposure for 48 h effectively reduces *C*Las content [17,18]. However, heat therapy cannot be widely used to control HLB. Plant growth regulators can improve the resistance of citrus to some extent and delay the course of HLB [18]. *Bacillus amyloliquefaciens* GJ1 was used to enhance photosynthesis and expression of resistance-related genes to improve the disease resistance of citrus [19]. Growth regulators and biological control cannot eliminate *C*Las, and it is not a long-term or effective strategy to control HLB. The use of chemical antibacterial agents is an effective means to control plant pathogens and increase yield [20,21]. Broad-spectrum antibiotics are often used to eliminate the symptoms of diseased trees. Penicillin, streptomycin, and oxytetracycline hydrochloride can effectively inhibit or even stop the growth of *C*Las and improve fruit quality through trunk injection or root soaking [22,23]. However, studies have shown that the excessive or unscientific use of antibiotics can lead to drug resistance in plants, and antibiotic control is unsustainable. If antibiotics are stopped, HLB may relapse within a few months. The development of drug resistance and the impact of antibiotics on human health limit the use of antibiotics [24,25]. There is still a lack of effective antibacterial agents for controlling HLB [26].

To develop an efficient and accurate method for screening agents against HLB, this study first detects the distribution of *C*Las in citrus plants. Based on the test results, a bioactivity test method is developed, and the feasibility of the method is evaluated. To find effective antibacterial agents against HLB, the antibacterial activity of sulfone compounds synthesized by our research group is tested on HLB [27,28]. We plan to work long-term on the discovery of novel antimicrobial agents against HLB using this method in the future.

## 2. Results

### 2.1. Identification of Samples Infected with HLB

**Figure 1:**
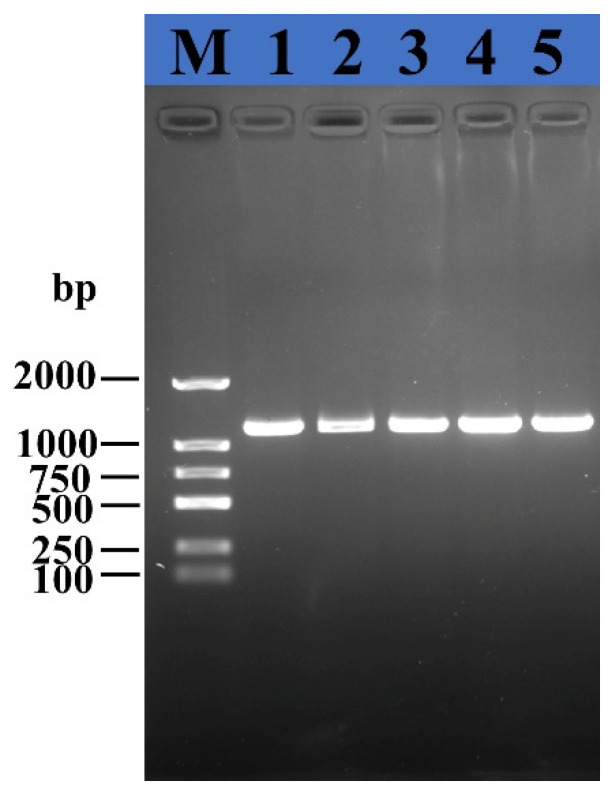
The PCR products of citrus leaves were electrophoretically amplified with OI1/OI2C primers. M, 2000 maker; 1–5, samples at different locations.

The results of gel electrophoresis showed that the amplified products from leaves collected from different positions had an obvious band structure between 1000–2000 bp, which was consistent with the literature reports (Figure 1). The homology of the PCR products and the NCBI website with *C*Las was 99.38%. According to the symptoms of the citrus trees and the detection results, most citrus trees in the orchards were infected with HLB.

### 2.2. Comparison of the Contents of CLas in Leaves

**Figure 2:**
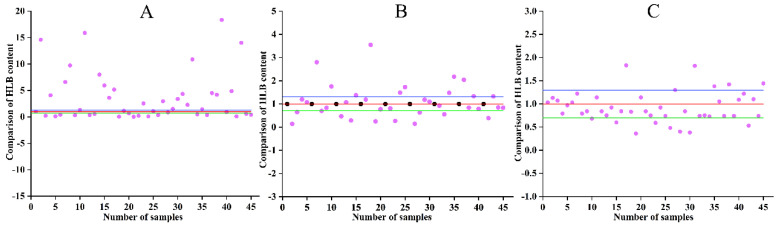
Comparison of *C*Las content in leaves. The red line indicates the same *C*Las content, and the green and blue horizontal lines indicate a deviation of 0.7–1.3 times. The purple dots represent the *C*Las content of each leaf. (**A**) *C*Las content in leaves from different branches; (**B**) *C*Las content in different leaves from the same branch (9 branches were taken, and the black dots represent the reference leaves of each branch); (**C**) *C*Las content in two halves of the same leaf.

To understand the difference in *C*Las content in citrus leaves, the *C*Las contents in leaves of different branches, different leaves of the same branch, and two halves of the same leaf (Left and right sides of symmetric blades) were tested, respectively, and each test was repeated 45 times (Figure 2). Through data analysis, it was found that the *C*Las contents of leaves of different branches, different leaves of the same branch, and two halves of the same leaf were significantly different. Among them, the difference in *C*Las content of leaves from different branches varied from several times to dozens of times. The difference in *C*Las contents of different leaves from the same branch decreased obviously, with a difference of about 0–3 times, while the difference in *C*Las content between two halves of the same leaf was small, at 0.7–1.3 times.

### 2.3. Design of Anti-HLB Activity Testing Method

**Figure 3:**
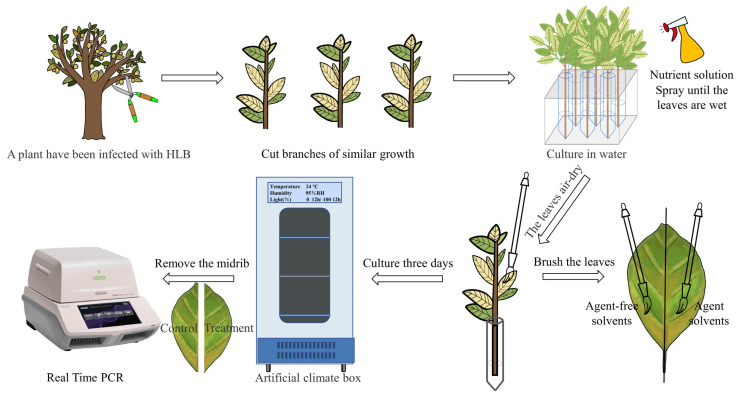
Half-leaf method for rapid screening of anti-HLB agents.

Based on the detection results of the *C*Las content in leaves, an indoor test method for resistance to HLB was designed (Figure 3), with the specific implementation mode as follows: (1) We collected branches infected with HLB in the field and evenly sprayed the nutrient solution to wet the leaves. (2) The right halves of the branches and leaves were evenly coated with the prepared medicine using a brush, and the left halves of the leaves were coated with the same curative solvent without medicine as the control (CK); the samples were then put into a thermostatic incubator for cultivation. (3) We sprayed the nutrient solution evenly every 24 h. (4) After 3 days, we took the branches out, picked three leaves randomly from each branch, and cut the leaves in two by halving leaves along the vein. (5) The left and right halves of the leaves were collected, wrapped in foil and labeled, and then frozen in liquid nitrogen and stored in a −80 °C refrigerator. (6) The relative proportions of the target gene in the treatment group and the control group were determined by quantitative Real-Time PCR (qPCR), and then the inhibitory activity of the agent was calculated.

### 2.4. Method Accuracy Verification

A total of 45 leaves were selected for 45 tests, and 6-chloropurine riboside was used as a testing agent. According to the results of *C*Las content in the citrus leaves, the deviation in *C*Las content in two halves of the same leaf was ±30%; the theoretical maximum activity and minimum activity were calculated, and the final activity was their average value (Table 1). The average of 45 groups of activity data was 17.95 ± 12.41%. The data were sequentially divided into five groups (each group was repeated nine times), and the average activity of each group was calculated to be 17.83 ± 12.65%, 17.29 ± 14.09%, 16.74 ± 11.90%, 19.26 ± 11.97%, and 17.04 ± 13.03%, respectively. From the results, although individual activity data were in the range of 0.24–45.35%, there was no significant difference among the five groups. The experimental results showed that nine leaves from three susceptible branches could be used as test plants to test the agent activity, and the test results were more accurate.

### 2.5. Determination of Anti-HLB Activity of Sulfone Compounds

**Figure 4:**
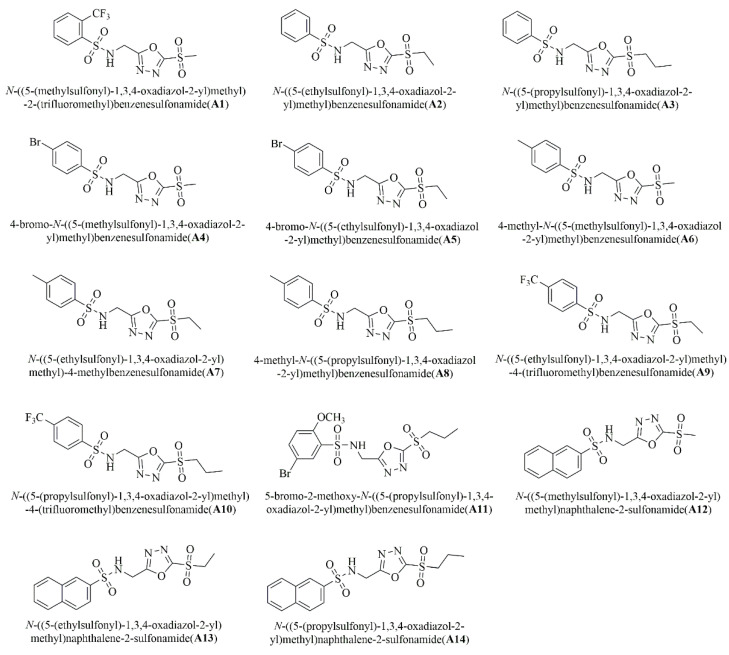
Chemical structures of sulfonamide compounds containing 1,3,4-oxadiazole sulfone moiety.

**Figure 5:**
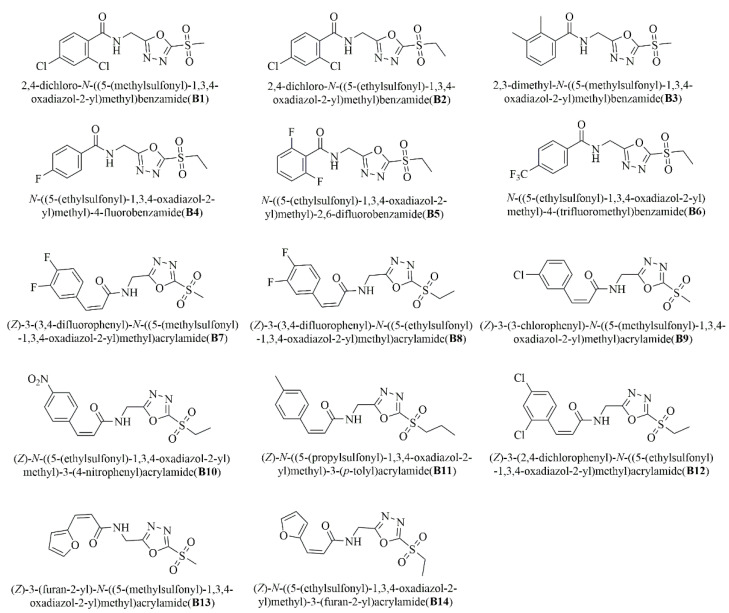
Chemical structures of amide compounds containing 1,3,4-oxadiazole sulfone moiety.

Our previous research found that some 1,3,4-oxadiazole sulfone compounds had excellent antibacterial activity against two plant pathogenic bacteria: *Xanthomonas oryzae* pv. *oryzicola* and *Xanthomonas oryzae* pv. *oryzae*. To confirm the feasibility of this method and to find a high-activity antibacterial agent against HLB, the activities of 1,3,4-oxadiazole sulfone compounds **A1–A14** and **B1–B14** against HLB were determined using the “half-leaf method” (Figure 4 and Figure 5). The deviations of all the antibacterial activity data were within 20%, which was consistent with the results verified by our method (Table 2). Encouragingly, some of the target compounds showed excellent activity against HLB. For example, the curative activities of compounds **A1** and **B6** were 76.3% and 70.0%, respectively.

The type of substituents of target compounds had a great influence on the activity against HLB. Compounds with trifluoromethyl or fluorine had better therapeutic activity, such as the two most active compounds, A1 and B6. On the other hand, the activity of short-chain alkanes connected with sulfone groups was better than that of long-chain alkanes, such as A12 > A13 > A14, B6 > B7 > B8.

## 3. Discussion

HLB caused by *C*Las is an unresolved global problem, causing a devastating blow to the global citrus industry. At present, *C*Las cannot be cultured in vitro, and there is no method that can quickly, accurately, and efficiently screen antibacterial agents against HLB. The screening of anti-HLB agents is usually performed by injecting, spraying, or soaking diseased trees in pots or fields, and the effects are evaluated by regular symptom observation or qPCR detection of *C*Las content [9,23,26]. These screening methods usually take several months or even years to obtain results, and due to the large differences in the contents of *C*Las in each leaf, the repeatability of the results obtained by sampling and detecting the changes in the *C*Las content in the leaves is often weak. Therefore, discovering a fast, accurate, and efficient method to test the antibacterial activity of an antibacterial agent against HLB is of great significance for the transition from prevention to treatment of HLB.

To discover the distribution of *C*Las content in leaves, the *C*Las contents of the leaves were tested. For example, the contents of *C*Las in leaves on different branches, different leaves from the same branch, and the left and right halves of the same leaf on the same branch were evaluated, respectively. To reduce the error caused by uneven distribution of *C*Las content on the experimental results, leaves with the same growth potential that were as symmetrical as possible were selected for testing anti-HLB activity, and all the veins in the middle of the leaves were removed when detecting the *C*Las content in the leaves. By comparing the *C*Las contents of two leaf halves, we were surprised to find that the difference in *C*Las content was in a relatively small range. We designed a method to screen the anti-HLB activity of antibacterial agents. Taking the vein as the boundary, half of the leaves were treated with antibacterial agents or compounds, and the other half of the leaves remained without treatment with antibacterial agents or compounds (CK). After several days, the *C*Las contents of the treatment group and the control group were detected, and the inhibition rate of *C*Las proliferation was obtained. In this way, the anti-HLB activity of each antibacterial agent and compound was calculated. The Cq value of CK should be less than 30, and a value greater than 30 indicated that the *C*Las content in the leaves was too low and the measurement error was large. In this study, a large number of test results showed that the deviation in *C*Las content of the two halves of the same leaf was ±30%. If the *C*Las content of the treatment group was 30% higher than that of the control group, the theoretical maximum activity could be calculated according to the difference in the final *C*Las content. On the contrary, if the *C*Las content of the treatment group was 30% lower than the control group, the theoretical minimum activity could be calculated according to the difference in the final *C*Las content. When the calculated activity value was negative, it was written as 0 (no activity), and we calculated the average value as the final activity. This calculation aimed to further reduce the error caused by the difference in *C*Las contents of the leaves.

## 4. Materials and Methods

### 4.1. Citrus Material

The citrus branch samples used in this study were collected from an orchard in Longping Town, Luodian County, Guizhou Province, China. The longitude and latitude were 106°77′ and 25°49′.

### 4.2. Analysis of Leaf Disease

Citrus leaves used for identification were randomly collected at five locations in the orchard. Then, the total DNA of the leaves was extracted using the CTAB method [29]. The 16s rDNA of *C*Las was amplified using PCR (forward primers 5′-GCGCGTATGCAATACGAGCGGCA-3′ and reverse primers 5′-GCCTCGCGACTTCGCAACCCAT-3′) [30]. The PCR procedure was as follows: 95 °C for 3 min, 95 °C for 30 s, 64 °C for 30 s, and 72 °C for 70 s for 35 cycles followed by 72 °C for 10 min. PCR products were analyzed with 1% agarose gel electrophoresis and Sangon Biotech Co., Ltd. (Shanghai, China). 

### 4.3. Comparison of Contents of CLas in Leaves

After selecting leaves with similar growth and symmetrical left and right sides, the midvein was removed from the leaves, and the left and right halves of the leaves were wrapped in foil, frozen in liquid nitrogen, and stored at −80 °C. 

We extracted the total RNA from the citrus leaves using the Trizol method [31]. The specific operation steps were as follows: The leaf samples were thoroughly ground to powder in liquid nitrogen and 50–100 mg was put into a 1.5 mL sterilized centrifuge tube. A total of 800 μL of Fruit-mate^TM^ was added, mixed well, and then left to rest for 5 min and centrifuged for 5 min (12,000 rpm, 4 °C). An amount of 500 μL of the supernatant was absorbed into a new sterilized centrifuge tube and mixed with 500 μL of trizol; 200 μL of chloroform was added after 5 min of standing. It was then left standing for 5 min after severe vibration and centrifugation were carried out for 15 min (12,000 rpm, 4 °C). A total of 500 μL of the supernatant was drawn into a new sterilized centrifuge tube, 500 μL of isopropanol was added, and the mixture was completely mixed upside down. The supernatant was placed on ice for 15 min, and then centrifuged for 10 min (12,000 rpm, 4 °C). We discarded the supernatant and added 800 μL of 75% ethanol to wash the precipitation, followed by centrifugation for 10 min (12,000 rpm, 4 °C). This was followed by pouring out the ethanol, centrifuging for 1 min (12,000 rpm, 4 °C), sucking out the residual water in the centrifuge tube, placing it at room temperature for 10 min, and adding 10 μL of sterilized enzyme water to dissolve the RNA. The extracted RNA was reverse-transcribed into cDNA, and the expression level of the target gene was detected using qPCR. Primers used were the *C*Las detection primer pair optimized by Li et al. (forward primer 5′-TCGAGCGCGTATGCAATACG-3′and reverse primer 5′-GCGTTATCCCGTAGAAAAAGGTA-3′) [32] and the internal reference using Citrus *Actin* (forward primer: 5′-GGTATTGCCGACCGTATGAG-3′ and forward reverse: 5′-TGGAAGGTGCTGAGGGATG-3′) [24]. Reaction conditions were as follows: 95 °C for 2 min, 95 °C for 20 s, 57 °C for 30 s, and 70 °C for 30 s for 40 cycles, with fluorescence signal reads at the end for 57 °C with each cycle. Three replicates of each sample were taken for the average Cq value. The expression of the target gene of *C*Las was obtained by calculating the value of 2^−ΔΔCq^. 

The *C*Las content mentioned in this study was the relative expression of genes. The *C*Las contents of different leaves from different branches and from the same branch were used to select a set of data (a leaf) as a control to calculate the relative gene expression. 

### 4.4. Method Validation

An amount of 5 mg of 6-chloropurine riboside was weighed into a 15 mL centrifuge tube, and we added 9.8 mL of 0.1% Tween-80 solution to prepare 500 mg/L of solution. Branches with similar growth were selected, cultivated in water, and sprayed with the nutrient solution evenly to wet the leaves. After the leaves were naturally air-dried, the right halves of the leaves were brushed with the prepared agent, and the left halves of the leaves were brushed with 0.1% Tween-80 as a blank control. We placed them in an artificial climate box and cultivated them for three days (25 °C, 95 RH), spraying the nutrient solution every 24 h to keep the leaves fresh. Three days later, the leaves of each treatment group and the control group were collected to analyze the expressions of the target gene of *C*Las to calculate the inhibitory activity.

### 4.5. Determination of Anti-HLB Activity of Sulfone Compounds

A total of 1 mg of compound was weighed, dissolved in 100 μL of DMSO, and added to 4.9 mL of 0.1% Tween-80 to prepare a 200 mg/L solution. A test solution without compounds was used as the negative control. The antibiotic bactericides of kasugamycin were used as positive controls. The treatment method of brushing the leaves and the activity calculation were the same as that of the method verification. Nine leaves from three closely growing branches were treated with each agent.

### 4.6. Statistical Analysis

The data are presented as the mean values ± standard error (SE) of nine replicates. The statistical analysis was performed using SPSS 23.0 software.

## 5. Conclusions

HLB seriously threatens the healthy development of the citrus industry in the world. At present, there is no effective antibacterial agent against HLB, and there is a lack of accurate and rapid screening methods for anti-HLB activity. In this research, we designed a method that could quickly screen the antibacterial activity of antibacterial agents against HLB. The accuracy, reliability, and repeatability of this method were validated using 6-chloropurine nucleoside. The activity data and bias obtained from the validation of the method with 6-chloropurine riboside and measured by antibacterially active sulfone compounds were in a relatively ideal range. It is recommended to use nine leaves from three branches (three leaves per branch) of the same tree as the test material to obtain more accurate activity data. This method of screening agents with citrus branches in the laboratory has the advantages of simple operation, a short cycle, and testing multiple agents at one time. The aim of this method is to screen substances with antibacterial activity from thousands of substances and to excrete substances without antibacterial activity, which can improve the screening efficiency.

## Figures and Tables

**Table 1:** Activity data for 6-chloropurine riboside against HLB.

Number	Maximum Activity %	Minimal Activity %	Activity %	Average *^a^*
1	35.38	0.00	17.69	17.83 ± 12.65 *
2	6.09	0.00	3.05	
3	44.46	0.00	22.23	
4	25.81	0.00	12.90	
5	16.91	0.00	8.46	
6	54.64	15.77	35.21	
7	40.55	0.00	20.27	
8	56.45	19.12	37.78	
9	5.73	0.00	2.86	
10	44.62	0.00	22.31	17.29 ± 14.09 *
11	54.30	15.13	34.72	
12	17.42	0.00	8.71	
13	0.48	0.00	0.24	
14	60.78	27.16	43.97	
15	25.84	0.00	12.92	
16	20.29	0.00	10.14	
17	31.67	0.00	15.83	
18	13.55	0.00	6.78	
19	61.75	28.96	45.35	18.63 ± 14.12 *
20	33.11	0.00	16.56	
21	48.04	3.51	25.78	
22	10.53	0.00	5.26	
23	49.93	7.01	34.28	
24	32.78	0.00	16.39	
25	28.27	0.00	14.14	
26	11.64	0.00	5.82	
27	8.16	0.00	4.08	
28	10.58	0.00	5.29	16.74 ± 11.90 *
29	5.64	0.00	2.82	
30	32.69	0.00	16.35	
31	28.63	0.00	14.31	
32	57.11	20.35	38.73	
33	52.30	11.42	31.86	
34	34.52	0.00	17.26	
35	14.55	0.00	7.28	
36	33.48	0.00	16.74	
37	5.18	0.00	2.59	19.26 ± 11.97 *
38	57.13	20.39	38.76	
39	52.64	12.04	32.34	
40	47.97	3.38	25.67	
41	30.20	0.00	15.10	
42	25.88	0.00	12.94	
43	9.96	0.00	4.98	
44	45.63	0.00	22.82	
45	36.36	0.00	18.18	
average *^b^*	31.55 ± 18.08	4.09 ± 8.04	17.95 ± 12.41	

*^a^* The average values of nine groups of activity data were obtained for nine leaves from three branches. *^b^* The average values of 45 groups of activity data. * The average values were no significant differences.

**Table 2:** Activity data of sulfone compounds against HLB.

Number	Activity (%)	Number	Activity (%)
A1	76.3 ± 2.6	B1	14.5 ± 11.5
A2	19.2 ± 3.6	B2	12.0 ± 10.5
A3	16.5 ± 7.5	B3	19.4 ± 6.5
A4	19.5 ± 16.6	B4	52.2 ± 9.3
A5	14.0 ± 13.9	B5	6.9 ± 13.5
A6	43.5 ± 5.5	B6	70.0 ± 6.0
A7	32.4 ± 4.7	B7	47.8 ± 7.4
A8	21.2 ± 8.1	B8	42.1 ± 13.9
A9	62.9 ± 3.4	B9	31.8 ± 7.9
A10	24.0 ± 7.5	B10	54.0 ± 11.9
A11	31.5 ± 6.5	B11	24.0 ± 10.9
A12	66.2 ± 10.8	B12	19.3 ± 5.2
A13	17.0 ± 8.3	B13	49.3 ± 8.4
A14	2.6 ± 10.2	B14	40.9 ± 4.7
kasugamycin	48.3 ± 14.0		

## Data Availability

The data that support the findings of this study are available from the corresponding author upon reasonable request.

## Abbreviations

HLB	Citrus Huanglongbing
*C*Las	*Candidatus* Liberibacter asiaticus
*C*Laf	*Candidatus* Liberibacter africanus
*C*lam	*Candidatus* Liberibacter americanus
ACP	*Diphorina citri* Kuwayama
qPCR	quantitative Real-Time PCR
